# Small Intestinal Bacterial Overgrowth in Subclinical Hypothyroidism of Pregnant Women

**DOI:** 10.3389/fendo.2021.604070

**Published:** 2021-05-24

**Authors:** Biao Wang, Yajuan Xu, Xiaofeng Hou, Jingjing Li, Yanjun Cai, Yingqi Hao, Qian Ouyang, Bo Wu, Zongzong Sun, Miao Zhang, Yanjie Ban

**Affiliations:** Department of Obstetrics and Gynecology, The Third Affiliated Hospital of Zhengzhou University, Zhengzhou, China

**Keywords:** hypothyroidism, pregnancy, breath test, intestine, microbiota

## Abstract

**Objective:**

To evaluate the small intestinal bacterial overgrowth (SIBO) of subclinical hypothyroidism of pregnant women, and explore their possible relevance.

**Methods:**

In total, 224 pregnant women with subclinical hypothyroidism during pregnancy (study group) and 196 pregnant women whose thyroid function was normal (control group) were enrolled in this study. Lactulose-based hydrogen and methane breath test was performed to evaluate the growth of intestinal bacteria. The serum-free thyroid hormone (FT4), thyroid-stimulating hormone (TSH), thyroid peroxidase antibody (TPOAb), body mass index (BMI) and gastrointestinal symptoms were detected and recorded.

**Results:**

The positive rates of SIBO were 56.7% and 31.6% in study group and control group, respectively. The levels of C response protein (CRP), abdominal distension and constipation in study group were higher than those in the control group. The risk of abdominal distension and constipation in SIBO-positive pregnant women were higher than that in SIBO-negative pregnant women, and the BMI of SIBO-positive patients in the two groups was lower than that of SIBO-negative patients in each group. In addition, the TPOAb-positive rate and TSH levels were higher but the FT4 level was lower in SIBO-positive patients compared to SIBO-negative patients in study group.

**Conclusion:**

The occurrence of subclinical hypothyroidism is related to SIBO, and the excessive growth of small intestinal bacteria may affect gastrointestinal symptoms.

**Clinical Trial:**

http://www.chictr.org.cn/index.aspx, identifier ChiCTR1900026326.

## Introduction

Hypothyroidism during pregnancy is an immune-related dysfunction occurring during pregnancy, affecting 3% - 5% of pregnant women (1), including overt hypothyroidism, subclinical hypothyroidism and isolated hypothyroxinemia. The main reason for the occurrence of hypothyroidism during pregnancy is that the demand for thyroid hormone of pregnant women and infants increases, while the synthesis and secretion of maternal thyroid hormone reduces. At this time, the risk of fetal nervous system development disorder is increased (2). Moreover, the risk of premature delivery, low birth weight, abortion, placental abruption and neonatal autism are also higher in clinical hypothyroidism and subclinical hypothyroidism (3, 4), and gastrointestinal related symptoms often occur at the same time (5). Therefore, it is important to further clarify the related factors.

Millions of microorganisms in the intestine constitute the intestinal flora, which interacts with the host and maintains the intestinal homeostasis of individuals (6, 7). When the small intestine is abnormally colonized by a large number of abnormal increased intestine bacteria, it is called small intestinal bacterial overgrowth (SIBO) (8), which affects the body’s intestinal function, endocrine and immune function. Most patients show a series of gastrointestinal symptoms, such as abdominal distension, constipation, diarrhea and even some systemic immune diseases, such as hypothyroidism, diabetes and hypertension (9). Human cells are unable to produce hydrogen and methane gases. When SIBO occurs, abnormal bacteria in the small intestine metabolize carbohydrates to produce hydrogen, methane and carbon dioxide, which are transported to pulmonary capillaries by passive diffusion and eliminated from the body, and the contents can be detected by chromatography (10). Lauritano et al. (11) performed the glucose-based hydrogen breath test on 50 patients with overt hypothyroidism and 40 patients with normal thyroid function, and they reported that the incidence of SIBO-positive patients in the overt hypothyroidism group was significantly higher than that in the control group. These authors also demonstrated that the incidence of abdominal distension in the SIBO-positive patients was significantly higher than that in the SIBO-negative patients. Although the relationship between overt hypothyroidism and SIBO has been reported, the evidence of SIBO in subclinical hypothyroidism is lacking, especially for pregnant women. To this end, we conducted this study.

## Materials and Methods

Clinical Trial Registration No: ChiCTR1900026326 Name: Study for intestinal bacterial overgrowth and complications during pregnancy. Participants: In total, 224 pregnant women with subclinical hypothyroidism were selected as the experimental group, and 196 normal pregnant women were selected as the control group. Inclusion criteria: The thyroid function level during pregnancy was detected by commercial kit (Roche, Shanghai, China), which was in line with the reference range formulated by the laboratory of the Third Affiliated Hospital of Zhengzhou University (subclinical hypothyroidism: 11.5 < FT4 < 22.7 pmol/L and TSH > 4.0 mIU/L; pregnant women with normal thyroid function: 11.5 < FT4 < 22.7 pmol/L, 0.4 < TSH < 4.0 mIU/L).

Exclusion criteria: Patients with the following characteristics were excluded: (1) age < 18 years old; (2) endocrine and immune-related pregnancy complications, such as diabetes, hypertension, systemic lupus erythematosus or hypothyroidism, which were diagnosed before pregnancy; (3) multiple pregnancies or fetal death during this pregnancy; (4) artificial insemination or assisted reproductive technology were used during this pregnancy; (5) obvious symptoms of stress, anxiety and depression; and (6) probiotics or antibiotics were used in the past three months as well as any form of treatment for gastrointestinal discomfort. All subjects were of Han nationality with similar diet, and all patients grew up and lived in Zhengzhou, Henan Province, China.

### Lactulose-Based Hydrogen and Methane Breath Test

All participants avoided using antibiotics for the last 4 weeks before the breath test, stopped taking gastrointestinal motility drugs and laxatives at least one week before breath test, avoided eating dairy products, bean products, sugared food, sugared drinks and fermented food rich in cellulose, such as wheat bread, pasta, cereals, vegetables and fruits, on the day before breath test and fasted for 8-12 hours before the breath test. All patients had no smoking history. During the breath test, all subjects were allowed to sit and rest as well as drink a small amount of water without sugar, but they were not allowed to eat any food. All patients drank 10 g of lactulose solution immediately after the first alveolar end expiratory test, and samples were collected on an empty stomach. Samples were collected every 20 minutes (12). Six samples were collected for each patient. All gases were detected by a Quintron Gas Chromatography system.

### Identification of Breath Test Results

The breath test results were analyzed as follows: (1) an increase of ≥20 ppm from baseline in hydrogen by 90 minutes was considered a positive test to suggest the presence of SIBO; (2) if the methane concentration was higher than the fasting baseline value by 10 ppm within 90 minutes of the breath test, SIBO was considered positive; and (3) if the hydrogen and methane concentration did not reach the above values, the sum of the two was higher than the sum of the fasting baseline values of hydrogen and the methane concentration was more than 15 ppm within 90 minutes of the breath test, SIBO was considered positive (12).

### Clinical Symptoms

We recorded the presence or absence of abdominal distension, diarrhea, abdominal pain, constipation and abdominal symptoms of all subjects. The constipation evaluation criteria (13) were as follows (two or more of the following six items): (1) straining during more than one-fourth (25%) of defecations; (2) lumpy or hard stools > 25% of the time; (3) sensation of anorectal obstruction > 25% of the time; (4) sensation of incomplete evacuation > 25% of the time; (5) manual maneuvers required to aid defecation > 25% of the time; and (6) fewer than 3 bowel movements per week. In addition to the above, the following three criteria were met to diagnose functional constipation: (1) loose stools rarely occurred when laxatives were not used; (2) constipation not sufficient to diagnose IBS; and (3) constipation lasting at least for 3 months within 6 months.

### Statistical Analysis

SPSS V24.0 was selected as the statistical analysis software. The enumeration data were statistically described by frequency and rate, and χ^2^ test was used for comparison between groups. The quantitative data of normal distribution were described by mean ± standard deviation (`x ± s). T test and rank sum test were used for comparison between groups. The difference was statistically significant when P < 0.05.

## Results

There was no significant difference in age, gestational week, weight, height, BMI and FT4 between study group and control group, but the TSH level in study group was higher than that in control group (**Table 1**). The plasma CRP level (**Table 1**), positive rate of SIBO, positive rate of hydrogen and methane in study group were significantly higher than those in control group. In addition, the number of patients with pure hydrogen-positive was greater than that of patients with pure methane-positive in both groups, and the difference was statistically significant (**Table 2**).

**Table 1:** Characteristics of Pregnant women with subclinical hypothyroidism (study group) and the control group (control group).

	study group	control group	*p*
Patients(Num)	224	196	–
Age(years)	30.93 ± 4.52	31.23 ± 4.77	0.507
Gestational weeks(weeks)	25.98 ± 1.33	26.17 ± 1.29	0.128
Height(cm)	162.27 ± 4.91	161.79 ± 4.66	0.312
Weight(kg)	59.06 ± 5.55	58.36 ± 5.46	0.193
BMI (kg/m2)	22.45 ± 2.04	22.29 ± 1.76	0.394
TSH (mIU/L)	5.69 ± 1.21	1.45 ± 0.56	**0.000**
FT4 (pmol/L)	13.78 ± 1.67	13.63 ± 1.53	0.328
TPOAb-positive (N)	43	0	**0.000**
WBC (109/L)	8.91 ± 1.96	8.79 ± 2.12	0.531
Neutrophils(109/L)	6.58 ± 1.70	6.53 ± 1.90	0.775
Percentage of neutrophils(%)	73.19 ± 5.89	73.90 ± 5.91	0.213
Hemoglobin(g/L)	117.58 ± 11.86	118.69 ± 11.10	0.323
CRP (mg/L)	7.83 ± 2.47	4.67 ± 2.87	**0.000**

**Boldface** indicates statistical significance.

**Table 2:** The rate of SIBO-positive, pure Hydrogen-positive, pure Methane-positive and Hydrogen-Methane positive between Pregnant women with subclinical hypothyroidism (study group) and the control group (control group).

	SIBO+	Hydrogen+	Methane+	Hydrogen/Methane+
study group	127(56.7%)	97(43.3%)	23(10.27%)	7(3.13%)
control group	62(31.63%)	53(27.04%)	6(3.06%)	3(1.53%)
*p*	**0.000**	**0.000**	**0.000**	**0.014**

**Boldface** indicates statistical significance.

The incidence rates of constipation and abdominal distension in the two groups were significantly higher than those of abdominal pain and diarrhea. The incidence of abdominal symptoms in study group was higher than that in control group, and the incidence of constipation in SIBO-positive patients in both groups was higher than those in SIBO-negative patients (**Table 3**). Although there was no significant difference in BMI between the two groups, the BMI of SIBO-positive patients in both groups was lower than that of SIBO-negative patients in each group, and the BMI of pure methane-positive patients was lower than that of hydrogen-positive subjects in each group with statistical significance (**Table 4**). The number of TPOAb-positive patients was 43 in study group, and 32 cases of which were SIBO-positive and 11 cases were SIBO-negative, and TPOAb was negative in control group. In addition, the FT4 level in study group was lower than that of control group, while the TSH level in study group was higher than that in control group (**Table 5**).

**Table 3:** The clinical abdominal symptoms of subclinical hypothyroidism group (study group) and the control group (control group), comparison of abdominal symptoms among SIBO-positive and negative patients in study group and control group, respectively.

		abdominal distension	abdominal pain	diarrhea	constipation
study group		89(39.7%)	28(12.5%)	21(9.3%)	97(43.3%)
control group		25(12.7%)	8(4.0%)	4(2.0%)	38(19.3%)
*p_s-c_*		**0.000**	**0.003**	**0.002**	**0.000**
study group	SIBO+	56	18	13	70
	SIBO-	33	10	8	27
*p_s_*		0.133	0.422	0.652	**0.000**
control group	SIBO+	14	4	3	22
	SIBO-	11	4	1	16
*p_c_*		**0.010**	0.268	0.094	**0.000**

**Boldface** indicates statistical significance.

p_s-c_: The p value of clinical abdominal symptoms between study group and control group.

p_s_: The p value of clinical abdominal symptoms between patients with SIBO-positive and negative in study group.

p_c_: The p value of clinical abdominal symptoms between patients with SIBO-positive and negative in control group.

**Table 4:** The comparison of BMI between SIBO-positive and negative, Hydrogen-positive, Methane-positive in pregnant women with subclinical hypothyroidism (study group) and the control group (control group), respectively.

BMI						
Group	SIBO+	SIBO-	*p*	Hydrogen+	Methane+	*p*
study group	21.38 ± 1.62	23.79 ± 1.77	**0.000**	21.60 ± 1.62	20.51 ± 1.55	**0.004**
control group	21.23 ± 1.47	22.78 ± 1.67	**0.000**	21.39 ± 1.51	20.38 ± 0.54	**0.004**

**Boldface** indicates statistical significance.

**Table 5:** The rate of TPOAb-positive and negative, FT4 and TSH levels in pregnant women with subclinical hypothyroidism (study group) and the control group (control group), respectively.

	SIBO+	SIBO-	*p*
TPOAb+	32	11	**0.01**
TPOAb-	95	86	
FT4	13.55 ± 1.61	14.09 ± 1.70	**0.017**
TSH	5.97 ± 1.37	5.32 ± 0.83	**0.000**

**Boldface** indicates statistical significance.

## Discussion

At present, the least expensive, noninvasive and widely used methods to detect SIBO are glucose hydrogen breath test (GHBT), lactose hydrogen breath test (LHBT) and D-xylose breath test (XBT). Human cells are unable to produce hydrogen and methane, which are only excreted from the lungs by carbohydrate metabolism and absorption by bacteria in the colon (14). However, glucose, the substrate of GHBT, is a monosaccharide, which is absorbed in the proximal small intestine, while lactulose, as a disaccharide, reaches the colon before being absorbed. This means that the LHBT can detect bacterial growth in the distal small intestine (15, 16). In addition, lactulose, as a substrate, does not cause blood glucose fluctuations in patients with diabetes, and it is not recommended to use glucose for detection for many pregnant women with blood glucose instability. Although XBT has high sensitivity and specificity, it may have a small amount of ^14^CO_2_ radioisotope release (17, 18), which is not suitable for pregnant women. Therefore, we used the LHBT to detect SIBO.

Subclinical hypothyroidism during pregnancy is a common disease in pregnancy, and its occurrence and development are closely related to immune and mechanism disorder. In recent years, the research on intestinal flora has increased. Intestinal bacterial metabolites, bacterial components and bacteria themselves constitute the intestinal flora signal and participate in the regulation of host immune defense and tolerance. *Yersinia enterocolitica* contain proteins associated with major thyroid antigens, which can affect thyroid function through a molecular simulation mechanism (19), and some particular bacteria such as Lactobacilli, some Bifidobacteria and *Candida albicans* may play an important role of potential pathogenic in autoimmune thyroid disease (20). Intestinal epithelial cells (IECs) form a physical and chemical barrier to isolate intestinal flora and host immune cells as well as to avoid excessive immune response and ensure the normal function of immune cells and immune organs (21, 22). In addition, short chain fatty acids (SCFAs), as metabolites of bacteria, affect intestinal barrier function and immune cell signaling pathways (23). SCFAs also mediate the differentiation of Th1, Th17 and CD4+ T cells that produce IL-10, and they affect the balance of the Th17/Treg ratio (24). Th17 promotes the occurrence of autoimmunity, while Tregs inhibit the excessive immune response. Many studies have found that there are differences between Th17 and Treg levels in peripheral blood of HT patients (25, 26).

Through the hydrogen and methane breath test, we found that the positive rates of hydrogen and methane in pregnant women with subclinical hypothyroidism during pregnancy were significantly higher than those in the control group. Compared to the control group, the structural composition and distribution of intestinal flora in the former group were disordered, and hydrogen and methane producing bacteria in the small intestine increased abnormally. Although the direct relationship with subclinical hypothyroidism has not been elucidated, intestinal flora still plays an indispensable role in the synthesis and utilization of thyroid hormone. Thyroid hormone is regulated by the negative feedback of hypothalamus, pituitary gland and thyroid gland. At the same time, somatostatin and dopamine also inhibit the release of TSH (27, 28), and these two factors can be mediated by microbiota (29). The maintenance of normal thyroid function requires the joint participation of trace elements, such as iodine and selenium, as well as deiodinase, glucuronic acid and bile acid (30), and intestinal flora plays an important role in the absorption and utilization of these trace elements and the regulation of enzyme activity (31, 32). In addition, some studies have found that the flora in the small intestine, cecum, colon and feces of rats can bind thyroxine reversibly, and antibiotic treatment can destroy this process (33). At the same time, the bioavailability of thyroxine depends on its ability to penetrate the intestinal barrier. Therefore, when small intestinal bacteria overgrowth occurs, the ecological balance of intestinal flora changes, which restricts the synthesis, secretion and utilization of thyroid hormone. Although the serum thyroxine level of subclinical hypothyroidism pregnant women was still in the normal range, we found that compared to the control group, the FT4 level of the SIBO-positive pregnant women with subclinical hypothyroidism was lower and that TSH was significantly higher than that of the SIBO-negative pregnant women. At the same time, the positive rate of TPOAb in SIBO-positive pregnant women with subclinical hypothyroidism was higher than that of the SIBO-negative pregnant women. These symptoms indicated that the excessive growth of intestinal bacteria would increase the risk of abnormal FT4, TSH and TPAB, suggesting potential further development of thyroid dysfunction.

Analysis of gastrointestinal symptoms of all subjects showed that subjects with subclinical hypothyroidism during pregnancy had a higher risk of constipation and abdominal distension than pregnant women with normal thyroid function. Previous studies have suggested that thyroid hormone affects intestinal motility by regulating the intestinal nervous system and changing smooth muscle function and gastrointestinal transit complex movement in the interdigestive period. However, many studies have shown that hypothyroidism is related to delayed gastric emptying, decreased intestinal peristalsis frequency and prolonged oral blindness time (34, 35), and these symptoms precisely point to the most common gastrointestinal symptom of hypothyroidism, namely constipation. Lauritano et al. found that patients with hypothyroidism are more likely to have abdominal distension when SIBO is positive, but they did not observe differences in constipation symptoms. Our study may have differed from previous research results due to regional diet and living habits. Lauritano et al. performed their study on overt hypothyroidism and did not investigate subclinical hypothyroidism. Therefore, the difference of disease classification and the particularity of pregnant women may play a greater role in the different abdominal symptoms.

Through analysis of the BMI of SIBO-positive and SIBO-negative subjects as well as the BMI of pure hydrogen-positive and methane-positive subjects, we found that BMI was significantly negatively correlated with the SIBO-positive rate and methane concentration. Thus, the positive rate of SIBO in obese pregnant women is lower, but the positive rate in emaciated women is higher. In this regard, we believe that when SIBO occurs, intestinal flora will be disordered and IECs will be damaged, thus affecting the individual’s absorption of carbohydrates, proteins and lipids as well as increasing the competition of nutrients due to the existence of bacteria (36). Specifically, *Methanobrevicter smithii* and *Methanosphaera stadtmanae* have been identified as the major methanogenic bacteria in human intestines (37). The former was initially thought to exist only in the colon, but rat models have shown that the bacteria are enriched throughout the small intestine. Although methanogens can interact with bacteria in nutrition and provide heat for the host (38), evidence from several large-scale studies supports the negative association between methanogens and obesity (39–41). In addition, methane slows down the intestinal transit time by 59% (42), and longer intestinal transport time, in turn, facilitates the growth of methanogens (43, 44). Therefore, the presence of these methanogens may also play an important role in constipation in SIBO-positive women with subclinical hypothyroidism during pregnancy.

Although the exact association between small intestinal bacteria overgrowth and subclinical hypothyroidism during pregnancy is not clear, dysfunctional flora and their metabolites may play a role through inflammatory and immune pathways. In this study, we found that the level of CRP in hypothyroid pregnant women was significantly higher than that in the control group. When the inflammatory response is strengthened, LPS induces activated macrophages to release inflammatory mediators, which bind to Toll-like receptor 4, initiating signal transduction, inducing IL-6 production, changing the function of regulatory T cells and disturbing the immune balance of the body (45). When the Th17/Treg ratio is unbalanced, thyroid follicles are destroyed, which restricts the normal performance of thyroid function.

In this study, by analyzing the exhaled gas of 420 subjects, we found that the incidence of small intestinal bacteria overgrowth in pregnant women with subclinical hypothyroidism was higher than that of pregnant women with normal thyroid function. In addition, the presence of methane and hydrogen in breath test was closely related to the occurrence of subclinical hypothyroidism in pregnancy, and the potential role of intestinal bacterial overgrowth in subclinical hypothyroidism during pregnancy was discussed. To the best of our knowledge, this is the first study to evaluate the intestinal bacteria of pregnant women with subclinical hypothyroidism by the lactulose-based methane and hydrogen breath test. However, as a cross-sectional study, there were several limitations in this study. There was a lack of immune indicators of subjects, and we did not longitudinally track the impact of gestational age factors on intestinal bacteria of pregnant women with subclinical hypothyroidism. Future studies will focus on the effect of treatment for small intestinal bacteria on the thyroid function and abdominal symptoms of patients, as this is a problem that we urgently need to solve.

## Data Availability Statement

The raw data supporting the conclusions of this article will be made available by the authors, without undue reservation.

## Ethics Statement

The studies involving human participants were reviewed and approved by the Ethics Committee of the Third Affiliated Hospital of Zhengzhou University. The patients/participants provided their written informed consent to participate in this study.

## Author Contributions

Conception and design: YjX and BW. Collection and assembly of data: BW, XfH, JjL, YjC, YqH, QO, BW. Data analysis and interpretation: BW, ZzS, MZ, and YjB. Manuscript writing: YjX and BW. All authors contributed to the article and approved the submitted version.

## Funding

This research was funded by Henan provincial science and technology research and development special funds #182102410020 (YjX).

## Conflict of Interest

The authors declare that the research was conducted in the absence of any commercial or financial relationships that could be construed as a potential conflict of interest.
